# Scraps

**Published:** 1886-09

**Authors:** 


### SCRAPS

PICKED UP BY THE ASSISTANT - EDITOR.

---

**Not such Bad Times after all.**—*The Medical Record* (New York) observes that now we have meat lozenges and meat suppositories, it may truly be said that the druggists' art has made both ends meat!

**Too Slow.**—In remarking upon the saying by Carlyle, that "dyspepsia kills poetic ambition," the *Boston Post* says: "If dyspepsia would only get its work in more constantly, it would be for the comfort of the world generally."

**Sir Thomas Watson and Sir James Paget.**—At the recent banquet of the British Medical Benevolent Fund, Dr. Broadbent, in proposing the health of the chairman, Sir James Paget, applied to him the words Sir James himself had used of Sir Thomas Watson—"His knowledge was so vast, his goodness so great, and his example so elevating, that we all wished he might spend part of his immortality on earth."

**Ilfracombe.**—Some idea of the popularity of Ilfracombe may be gathered from the fact that a census taken of the number of visitors who slept in the town one Sunday night in August shows the following result: Staying at hotels, 503; boarding-houses, 554; apartments, 3381; with friends, 150; or in all, 4588. It is calculated that during the month of August, about 2000 day excursionists visit the town every week by the South Western Railway alone. On one Sunday there was not standing room in the parish church.

**Hopeless.**—Sir Astley Cooper relates the following anecdote of an Irish candidate before the Examining Board of the London College:—"What is a simple and what is a compound fracture?" asked the examiner. The reply was: "A simple fracture is when a bone is broke, and a compound fracture when it's all broke." Sir Astley asked him what he meant by "all broke." "I mean," he replied, "broke into smithereens, to be sure." I ventured to ask him what was 'smithereens.' He turned upon me with an intense expression of sympathy upon his countenance, "You don't know what is smithereens? Then I give you up!"—Sir Charles A. Cameron in *Dublin Journal of Medical Science.*

**Boils.**—A boil is generally very small at first, and a fellow hardly notices it; but in a few days it gets to be the biggest of the two, and the chap that has it is of very little account in comparison with his boil, which then "has him." Boils appear mysteriously upon various portions of the human body, coming when and where "they darn please," and often in very inconvenient places. . . . They are generally very lively and playful at night, and it is very funny to see a chap with a large one prospecting around his couch, for a place where his boil will fit in "without hurting it." . . . It is said that boils save the patient a fit of sickness. It is also said that a person is better after he has them; and there is no doubt that one feels much better after having got rid of them.—*Physician and Pharmacist (Cincinnati Lancet and Clinic).*

**Lucre.**—An Indian physician was holding forth the other day to some of his brothers of the craft in England. "You sairs in the West," he said,

"do not understand the practice of medicine. In my country, if a rajah with nothing of sickness sends for me, I go and I say, 'Sair, your case is a bad one; you will be worse before you are better.' I give him some medicine and I go away. The next day I go again, and I find him heaving like a sea-sick mandarin, and wishing that the son of his mother had never seen the light. 'Sair,' I say, 'I told you so; you have passed a great crisis. There is no more need of medicine. Another sun will see your cure complete.' I then collect my fees, and I go away. When I have cured a few more rajahs, I shall come again to your country and take a villa on your little river Thames, with the green turf sloping down to the waterside."

**Ladies' Dress.**—The inevitable extremes of fashion have led, during the past winter, to that peculiar folly by which society women have aimed to display a minimum of dry goods upon a maximum of bust: the result has been that a fashion plate is but little different from an anatomical plate. It is the result, we suppose, of reaction from that dictum of society, or of *court* (as not long ago in Canada), which led women to dress as if they were affected with cynanche tonsillaris. They are in a fair way now to acquire that which they were then well protected against. A doctor who had just come from a dinner party was recently asked how the ladies were dressed. He replied, "Well, now, I give it up; I didn't look under the table."

In all ages of the civilised world satirist-poets have sung, moralists have preached, and physicians and hygienists have protested, concerning the dangers which each saw menacing the transgressors of the natural law of dress, and all, so far as one can see, absolutely without effect. The demands of fashion are like the religious prejudices of the ignorant fanatic—too strong to be altered either by persecution or ridicule. Of these two weapons we deem the latter the stronger; and we have been not a little surprised at seeing it assumed, on the offensive, in the columns of that staid and conservative contemporary of ours, the *Boston Medical and Surgical Journal:*

"To the medical man of serious tastes, who never, in whatever circumstances of frivolity he may find himself, forgets that he belongs to the gravest of professions, and who lets slip no opportunity of perfecting himself in the principles of the art, the prevailing mode of ladies' evening toilettes offers great possibilities.

"Dragged, perhaps unwillingly, from his sterner duties by the demands of social life, his time is yet not thrown away. Should surgery be his delight, he finds abundant facilities in the drawing-room or at the opera for viewing or reviewing topographical anatomy. The triangles of the neck are often more freely demonstrated by the very class of subjects in whom the anatomical landmarks are most clearly visible. There is more difficulty in studying the axillary space in its completeness, though its anterior and posterior boundaries, the pectoralis major and the latissimus dorsi, can usually be well made out. The advance of fashion has, unfortunately, not as yet brought under observation the other regions of great anatomo-topographical interest. While mechanically paying out his share of the small coin of social converse, the surgeon is really making the incisions for ligature of the subclavian, the brachial, and the innominate; he is considering how the appearances would be changed in case of a fracture of the coracoid process, or a dislocation of the humeral head, or of the sternal end of the clavicle. Perhaps he is planning out an operation for the ablation of the mammary gland. Who knows?

"If chest diseases are his specialty, he lays out the boundaries of the pulmonary lobes, wonders if the cardiac dullness extends further to the left than the line which his practiced eye describes as the normal boundary, and upon the lady who stands in front of him, determines the proper point to enter a needle for thoracentesis. These opportunities are not to be lightly overlooked by any."

We wonder if a single lady could be frightened out of her customary anatomical display by the thought that some doctor was planning out upon *her* fair form the operation he was the next day to perform upon some poor wretch; in other words, was utilizing the animated manikin thus presented to his gaze.—*The Medical Press of Western New York.*

